# The Spt-Ada-Gcn5 Acetyltransferase (SAGA) Complex in *Aspergillus nidulans*

**DOI:** 10.1371/journal.pone.0065221

**Published:** 2013-06-07

**Authors:** Paraskevi Georgakopoulos, Robin A. Lockington, Joan M. Kelly

**Affiliations:** School of Molecular and Biomedical Science, University of Adelaide, Adelaide, South Australia, Australia; Universidade de Sao Paulo, Brazil

## Abstract

A mutation screen in *Aspergillus nidulans* uncovered mutations in the *acdX* gene that led to altered repression by acetate, but not by glucose. AcdX of *A. nidulans* is highly conserved with Spt8p of *Saccharomyces cerevisiae*, and since Spt8p is a component of the Spt-Ada-Gcn5 Acetyltransferase (SAGA) complex, the SAGA complex may have a role in acetate repression in *A. nidulans*. We used a bioinformatic approach to identify genes encoding most members of the SAGA complex in *A. nidulans*, and a proteomic analysis to confirm that most protein components identified indeed exist as a complex in *A. nidulans*. No apparent compositional differences were detected in mycelia cultured in acetate compared to glucose medium. The methods used revealed apparent differences between Yeast and *A. nidulans* in the deubiquitination (DUB) module of the complex, which in *S. cerevisiae* consists of Sgf11p, Sus1p, and Ubp8p. Although a convincing homologue of *S. cerevisiae* Ubp8p was identified in the *A. nidulans* genome, there were no apparent homologues for Sus1p and Sgf11p. In addition, when the SAGA complex was purified from *A. nidulans*, members of the DUB module were not co-purified with the complex, indicating that functional homologues of Sus1p and Sgf11p were not part of the complex. Thus, deubiquitination of H2B-Ub in stress conditions is likely to be regulated differently in *A. nidulans* compared to *S. cerevisiae*.

## Introduction

The SAGA complex has been extensively studied in *Saccharomyces cerevisiae*, and is highly conserved from yeast to humans [1].

In *S. cerevisiae*, the SAGA complex is a 1.8 MDa multiprotein complex involved in the regulation of genes that are expressed in response to stresses including metabolic starvation, DNA damage and heat, that account for approximately 10% of yeast genes [2]. Certain components of the SAGA complex bind directly to the TATA-box binding protein (TBP), and these interactions are important for the recruitment of TBP to the promoter [3]. The SAGA complex in *S. cerevisiae* consists of approximately 20 polypeptide subunits, which form modules within the complex [4]. One module, required for histone acetyltransferase (HAT) activity, comprises Gcn5p, Ada2p, Ada3p and Sgf29p. A second module, required for TBP binding, contains both Spt3p and Spt8p. Mutations in genes encoding proteins in the third module, Spt20p, Spt7p and Ada1p, have severe phenotypes due to complete disruption of the SAGA complex, whilst mutations of Spt3p and Spt8p have milder phenotypes [5], [6]. The fourth module includes Ubp8p, a histone deubiquitinase, Sgf11p and Sus1p, which are co-dependent for the deubiquitinating activity and their interaction with the SAGA complex, and Sgf73p, which is required to maintain proper histone ubiquitination levels by anchoring the deubiquitination module to the SAGA complex [7]–[9]. SAGA also contains transcription association factors (TAF_II_s), Tra1, which interacts with activators, Chd1p, which is a chromatin remodeling protein that has been shown to specifically interact with methylated lysine 4 on Histone H3 that is associated with transcriptional activity, and Sgf29p, which binds to methylated histone H3K4, this in turn facilitates histone H3 acetylation by the SAGA complex [10]–[15].

Less is known about the SAGA complex in *A. nidulans*. Reyes-Dominguez et al. (2008) analysed strains containing deletions in *gcnE* (*GCN5*) and *adaB* (*ADA2*), and found that nucleosome positioning and histone H3 acetylation are independent processes at the *prnD-prnB* bi-directional promoter [16]. In inducing-repression conditions, *gcnE* and *adaB* deletion strains showed partial derepression of the *prnD-prnB* transcripts, indication the possible requirement of GcnE and AdaB for repression via CreA, which was surprising as Gcn5p and Ada2p are required for transcription of Gcn5p dependent promoters in *S. cerevisiae.* Neither deletion affected the fully induced levels of the *prnD-prnB* transcripts, suggesting that induction is independent of GcnE and AdaB [16]. When *A. nidulans* and *Streptomyces rapamycinicus* interact, both exhibit a stress response. Nuetzmann and colleagues used strains containing deletions in *gcnE* and *adaB* to investigate the response to bacterial stress in these *A. nidulans* strains, and showed that the bacterium induces a histone modification via the SAGA complex, and the activation of a cluster of genes required for the biosynthesis of secondary metabolites derived from orsellinic acid [17].

Carbon catabolite repression is a mechanism in microorganisms, which has evolved to regulate gene expression in response to their environment. In the presence of a favorable carbon source (e.g. glucose) the transcription of genes encoding enzymes required for the utilization of alternative carbon sources is repressed [18], [19].

In *Aspergillus nidulans*, glucose repression has been extensively studied, and repression of a large number of genes subject to carbon catabolite repression requires the transcriptional repressor CreA [20]. In *A. nidulans*, acetate is a repressing carbon source that leads to similar levels of CreA mediated repression as glucose [21]. The *acdX* gene was identified in a mutation screen in *A. nidulans* to identify mutations affecting acetate repression, but not glucose repression. The conservation of the amino acid sequence of AcdX of *A. nidulans* and the SAGA component Spt8 of *S. cerevisiae* initially suggested that the SAGA complex may play a role in acetate repression in *A. nidulans* [21].

Although some experiments on GcnE and AdaB null strains have been reported, no studies have previously been undertaken to show whether all the components of the SAGA complex are present in the *A. nidulans* genome, and whether the proteins form a complex in *A. nidulans*. We report results of bioinformatic analyses to indicate whether genes encoding the SAGA complex proteins are present in the *A. nidulans* genome. We followed this up using a biochemical approach, involving TAP-tag purification and western blot, to confirm which proteins are present as a physical complex in *A. nidulans*. Since initial studies indicated that *acdX* mutations affect acetate but not glucose repression [21], we also used protein purification to determine whether there are differences in protein composition of the SAGA complex in cells grown in glucose compared with acetate repressing conditions.

## Materials and Methods

### Bioinformatics Tools

The *Saccharomyces* genome database (http://www.yeastgenome.org/) and the *Aspergillus* genome database (http://www.aspgd.org/), which provide integrated biological information for the organisms, as well as tools for analysis and comparison of sequences, were used in this analysis. The Pairwise Sequence Alignment tool, EMBOSS Needle (http://www.ebi.ac.uk/Tools/psa/), which creates an optimal global alignment of two sequences using the Needle-Wunsch algorithm, was used to align sequences.

### Strains and Media

The genotypes of *A. nidulans* strains are shown in Table 1. *Aspergillus* complete and minimal media are based on those described by Cove [22]. Carbon and nitrogen sources were added aseptically to the media to the final concentrations shown for each test. Transformation of *A. nidulans* was based on the procedure of Tilburn et al. [23].

**Table 1:** Genotypes of strains used in this study.

Pseudonym	Genotype	Derivation
*acdXΔ;nkuAΔ*	*yA1;[acdX::A.f. riboB]; pyroA4 [nkuA::argB]; riboB2*	[21]
*^MYC^acdX;nkuAΔ*	*yA1;[A.f. riboB::^MYC^acdX];pyroA4 [nkuA::argB]; riboB2*	[21]
*sptCΔ;^ MYC^acdX;nkuAΔ*	*yA1;[^MYC^acdX];pyroA4[nkuA::argB];[sptC::A.f. riboB] riboB2*	[21]
*^N^* ^**−***TAP*^ *sptC;^MYC^acdX;nkuAΔ*	*yA1;[^MYC^acdX];pyroA4[nkuA::argB];[^N^* ^**−***TAP*^ *sptC]*	This work

### Construction of ^N**−**TAP^SptC Strain

To obtain a strain expressing SptC N-terminally epitope tagged with the tap tag (^N**−**TAP^SptC), a construct was made that contained ^N**−**TAP^ *sptC*. To achieve this, primers were designed to amplify N-TAP from pME2968 kindly provided by Professor Gerhard H. Braus [24]. These primers were designed to incorporate sites for the restriction enzymes *Nco*I and *Apa*I, to enable the desired vector to be obtained via a digestion/ligation approach (Table 2). Primers were also designed to amplify the vector containing *sptC* (pSPTC), such that the restriction sites for *Nco*I and *Apa*I were incorporated immediately after the start codon, such that upon ligation with the purified N-TAP PCR product, N-TAP would be incorporated immediately after the start codon and in frame (Table 2). The p^N**−**TAP^SPTC construct was linearized and transformed into a strain containing a deletion of *sptC* (*sptCΔ;* ^MYC^ *acdX;nkuAΔ*) [21]. Transformants were obtained by homologous integration as the strains used were in a *nkuAΔ* background [25], and detected by morphological observation.

**Table 2:** Oligonucleotide primers used in this study.

Primer name	Primer sequence 5′–3′
SptCfApaI	GAA GGG CCC TCG TCT GAT CGT ACT CCT
SptCrNcoI	GCT CCA TGG CAT ATT GCG ATT GCG AAT CTG GGA
N-TAPfNcoI	GCG CCA TGG GCC GTG GAC AAC AAA TTC
N-TAPrApaI	AGC GGG CCC ATC AAG TGC CCC GGA GGA

### Protein Purification and Tandem Affinity Purification (TAP) for *A. nidulans*

Tandem affinity purification was performed as described in [24]. The purified proteins were separated by polyacrylamide gel electrophoresis, and bands were excised from the silver-stained gel manually, then washed, destained, reduced, alkylated, digested, and extracted. Vacuum concentrated samples were resuspended with 0.1% FA in 2% ACN to a total volume of 8 µl. LC-eSI-IT MS/MS was performed using an online 1100 series HPLC system (Aligent Technologies) and HCT Ultra 3D-Ion-Trap mass spectrometer (Bruker Daltonics). The LC system was interfaced to the MS using an Agilent Technologies Chip Cube operating with a ProtlD-Chip-150 (II), which integrates the enriched column (Zorbax 300 SB-C18, 150 nm×75 nm), and nanospray emitter. Ionizable species were trapped and the two most intense ions were eluting at the time were fragmented by collision-induced dissociation. MS and MS/MS spectra were subjected to peak detection and de-convolution using DataAnalysis (Version 3.4, Burker Daltonics). Compound lists were exported into BioTools (Version 3.1, Burker Daltonics) then submitted to Mascot (Version 2.2).

## Results and Discussion

### Presence of Homologues of SAGA Complex Proteins in *A. nidulans*

Bioinformatic analysis was initially undertaken to indicate whether the SAGA complex components were present in the *A. nidulans* genome. The SAGA complex components required for structural integrity, HAT activity, TBP-binding, activator interaction, chromatin remodeling, and the TAF_II_s, were all present in the *A. nidulans* genome (Table 3). Although potential homologues of Ubp8p and Sgf73p, which in *S. cerevisiae* are part of the deubiquitinating module, were identified in the *A. nidulans* genome, there were no convincing Sus1p and Sgf11p homologues. Accession number AN7253 and AN8685 were most similar to Sus1p and Sgf11p respectively, and using EMBOSS needle alignment, Sus1p and AN7253 were 10.4% identical, and Sgf11p and AN8685 were 8.3% identical (Table 3). The Expect (E) values of Sus1p and Sgf1p are of the order E**^−^** ^.02^, whereas other proteins are very much lower E**^−^** ^.08^, and it is unlikely that these are homologues of the *S. cerevisiae* proteins.

**Table 3:** The SAGA complex components present in the *A. nidulans* genome.

Functional module^a^	*S. cerevisiae* b	*A. nidulans* Acc #^c^	Similarity/Identity	E-value
Structural integrity	Spt20p	AN0976 (RfeE)	22.1/13.4	1.0E**^−^** ^08^
“	Spt7p	AN4894	39.7/25.0	1.0E**^−^** ^89^
“	Ada1p	AN10953	35.1/22/7	2.0E**^−^** ^17^
Hat activity	Gcn5p	AN3621 (GcnE)	69.5/53.0	2.0E**^−^** ^135^
“	Ada2p	AN10763 (AdaB)	57.7/41.7	1.0E**^−^** ^106^
“	Ada3p	AN0440	39.1/24.2	1.0E**^−^** ^43^
TBP binding	Spt8p	AN4670 (AcdX)	44.9/29.2	4.0E**^−^** ^46^
“	Spt3p	AN0719 (SptC)	65.4/47.1	2.0E**^−^** ^75^
TAF_II_s	Taf5p	AN0292	42.0/28.2	8.0E**^−^** ^115^
“	Taf6p	AN8232	57.2/37.6	1.0E**^−^** ^90^
“	Taf9p	AN0794	35.1/24.3	3.0E**^−^** ^30^
“	Taf10p	AN0154	36.9/27.2	4.0E**^−^** ^14^
“	Taf12p	AN2769	37.3/24.1	1.0E**^−^** ^28^
Deubiquitination	Sgf73p	AN11747	24.4/16.1	1.0E**^−^** ^17^
“	Ubp8p	AN3711	44.9/30.7	1.0E**^−^** ^56^
“	Sus1p	AN7253	21.3/10.4	3.8E**^−^** ^02^
“	Sgf11p	AN8685	13.8/08.3	9.2E**^−^** ^02^
Interact H3K4^m^	Chd1p	AN1255	51.3/37.6	0.00
“	Sgf29p	AN0668	27.4/19.3	1.0E**^−^** ^23^
Interact activators	Tra1p	AN8000	22.1/13.4	0.00

a Functions of the *S. cerevisiae* SAGA complex subunits.

b *S. cerevisiae* homologues identified in *A. nidulans.*

c *A. nidulans* accession number.

References: RfeE [38], AdaB and GcnE [16], AcdX and SptC [21].

### Functional Expression of SptC Epitope Tagged with the Tandem Affinity Purification (TAP) Tag

**Figure 1:**
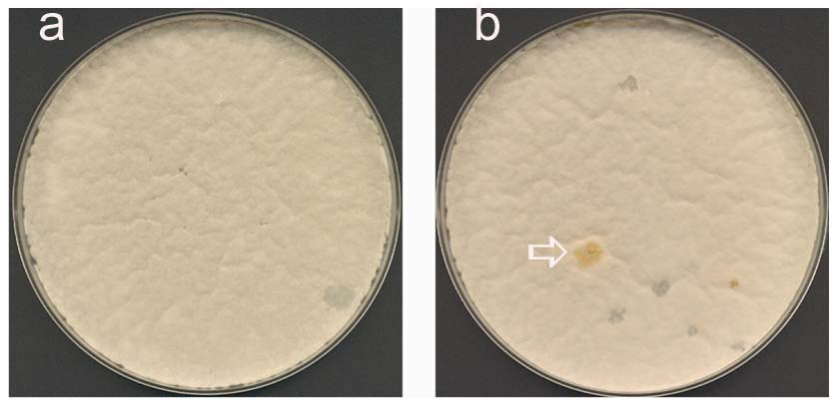
Complementation of the *sptCΔ* ^MYC^ *acdXnkuAΔ* by p^N−TAP^SPTC. *sptCΔ* ^MYC^ *acdXnkuAΔ* protoplasts plated on osmotically stabilised minimum medium, after 3 days growth at 37°C. **A)** No DNA control. **B)** Transformed with p^N**−**TAP^SPTC; arrow indicates complemented transformant.

To determine that the SAGA proteins exist as a complex in *A. nidulans*, SptC, the homologue of Spt3p known to be a component of the SAGA complex in *S. cerevisiae*, was epitope tagged with the TAP tag, to allow tandem affinity purification of the complex. The ^N**−**TAP^ *sptC* fusion was integrated into the *A. nidulans* genome as a single copy at its native locus, in a strain containing a deletion of *sptC*. Since *sptC* mutant strains conidiate poorly giving them a white appearance [21], (Figure 1a), this allows direct identification of complementing transformants, as if ^N**−**TAP^SptC is functional, all transformants should have strong, yellow conidiation. The desired transformants were obtained by homologous integration in an *nkuAΔ* background (Figure 1b), [25], and the presence of the N-TAP tag was confirmed (Figure S1).

**Figure 2:**
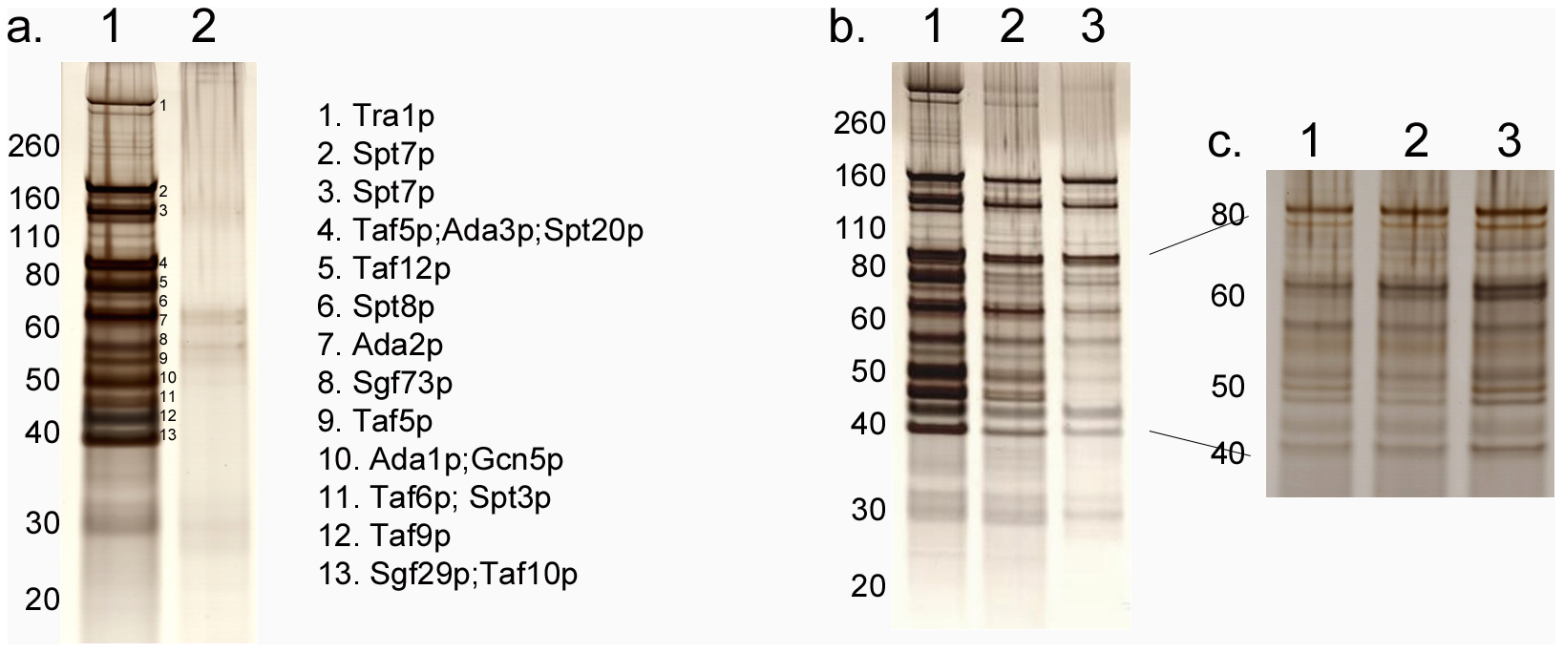
SAGA complex purification. a) Tandem affinity purification of a strain containing SptC tagged with the TAP tag (Lane 1) and a strain with wildtype SptC (Lane 2). The gel regions that were purified are numbered, and the *S. cerevisiae* homologues of the SAGA complex components identified in *A. nidulans* by LC MS are shown on the right. b) Tandem affinity purification of the N-TAP*sptC*;MYC*acdX*;*nkuAΔ* strain grown in media containing either 1% glucose (Lane 1), 50 mM arabinose (Lane 2) or 50 mM sodium acetate pH 6.0 (Lane 3). LC-MS was performed for all three conditions in this experiment. c) Figure 2c shows one of a further two repeat experiments, designed specifically to determine whether the differences in staining intensity around 50KDa in lane 3 of Figure 2b were robustly repeatable, showing that the apparent differences in part b are an artifact.

Initially, the SAGA complex was purified from strains grown in medium containing 1% glucose as the sole carbon source and 10 mM ammonium tartrate as the nitrogen source. Figure 2a shows the results of a silver-stained polyacrylamide gel, which contains the purified eluates of the experimental strain containing ^N**−**TAP^SptC, and the control strain containing wildtype SptC (Figure 2a). Gel slices containing proteins from the silver-stained gel were digested using trypsin, and peptides were analyzed by LC MS analysis. The control lane, containing the ^MYC^ *acdX; nkuAΔ* eluate, showed typical weak background bands from TAP purification, but no SAGA subunits were detected. The experimental lane, containing the ^N**-**TAP^SptC eluate, shows multiple bands, and LC MS analysis indicated that they were SAGA complex subunits. Figure 2a shows the SAGA complex subunits identified. Full details showing the complex components, *A. nidulans* accession numbers, predicted molecular weights, sequence coverage and the peptides identified are available in Table S1.

### SAGA Complex Protein Composition of *A. nidulans* in Carbon Repressing and Non-repressing Conditions

The purification was repeated using *A. nidulans* strains grown in medium containing 1% glucose, and was also performed in *A. nidulans* strains grown in media with either acetate or arabinose as the sole carbon source, to determine whether there were compositional differences of the SAGA complex between these growth conditions. LC MS analysis was performed for all growth conditions tested. Acetate was used as it has previously been shown to be a repressing carbon source in *A. nidulans*, and initial studies had indicated that components of the SAGA complex might have a role in acetate repression [21], and arabinose was used as the non-repressing carbon source. Figure 2b shows that there were no compositional differences for SAGA between the different carbon sources used. This result is consistent with findings that *acdX* mutations do not lead directly to transcriptional derepression in mycelia grown in acetate medium [21].

### Similarities and Differences of the SAGA Complex in *A. nidulans* and *S. cerevisiae*

In the growth conditions tested, *A. nidulans* was shown to contain the majority of the SAGA complex components seen in *S. cerevisiae*; however, the Ubp8p, and Chd1p homologues were not detected. Published microarray evidence indicates that AN3711 (*UBP8*), and AN1255 (*CHD1*) are expressed in glucose medium and the expression does not change in ethanol medium or in response to hypoxic conditions [26], [27]. *S. cerevisiae* Ubp8p is a histone H2B deubiquitinating enzyme that specifically removes monoubiquitin from lysine 123 of the H2B C-terminal tail [7], [28], and has been shown to form a distinct module within the SAGA complex with Sgf11p and Sus1p, which, like Ubp8p, are not required for the structural integrity of the SAGA complex. Sgf11p is required for the Ubp8p association with the SAGA complex and therefore H2B deubiquitination [29]. Furthermore, association of Sus1p with SAGA requires Ubp8p and Sgf11p. Loss of Sus1p causes an increase in H2B ubiquitinaton and H3 methylation, to similar levels as in strains lacking Ubp8p and Sgf11p. These results indicate that all three proteins are co-dependent for their interaction with SAGA, and therefore form a distinct module within the SAGA complex [8]. In the bioinformatic analysis clear *A. nidulans* homologues of the Sus1p and Sgf11p proteins were not identified with any confidence. AN7253 and AN8685, the most similar proteins to Sus1p and Sgf11p respectively, were not detected in the purified complex, providing further evidence that they are not functional homologues. Since Ubp8, Sus1p and Sgf11p are co-dependent for their interaction with the SAGA complex, through Sgf73 [9], the distinct deubiquiting module containing Ubp8p present in the SAGA complex in *S. cerevisiae* is most probably absent in the *A. nidulans* complex. Supporting this conclusion, the procedures used, Tap-tag purification followed by western blot, have routinely been used in Yeast in experiments where Ubp8 and Sus1 proteins were detected as part of the SAGA complex. For example, Henry and colleagues identified Ubp8 among Ada2-Tap-tag purified proteins, showing that Ubp8 is a stable component of the transcriptionally relevant SAGA and SALSA/SLIK complexes [28]. Rodriguez-Navarro and colleagues showed that Tap-tagged Sus1 enriched all members of the SAGA complex, and vice versa Tap-tagged SAGA subunits co precipitated Sus1 [30]. Pray-Grant and others identified Ubp8 among the proteins in a highly purified yeast SLIK complex [12]. And Kohler and colleagues used recriprocal Ada2-Tap tag and Sus1Tap-tag purifications, to show Sus1, Sgf11, and Ubp8 association with SAGA [8]. Thus if deubiquiting module components were present in the SAGA complex in *A. nidulans*, these methods should detect them.

AN3711 (Linkage Group II) encodes the most similar protein in the *A. nidulans* genome to the *S. cerevisiae* SAGA complex component Ubp8p. A deletion was made in a *nkuA*Δ strain of *A. nidulans* [25], and was phenotypically similar to wildtype [21]. This is consistent with the situation in yeast, where a *UBP8* deletion strain does not have a marked phenotype [28], due most probably to other proteins that can deubiquitinate histones [21].

Although there was a clear Chd1p homologue in the genome of *A. nidulans,* in the growth conditions tested, it was not detected in the SAGA complex in these analyses. In *S. cerevisiae*, Chd1p functions in chromatin remodeling, gene expression and transcriptional elongation [31]–[33]. In strains lacking Chd1p, there is a defect in the histone acetyltransferase activity (HAT) of SAGA on nucleosomal histones [12]. Chd1p contains two chromodomains, and of these chromodomain 2 facilitates SAGA HAT activity by interacting with methylated H3-Lys9 [12]. Transcriptionally inactive euchromatin is methylated on histone H3 at Lys 4, Lys9 and Lys 27 [34]. In *S. cerevisiae*, methylation of H3K4 at the *GAL10* locus, is tightly regulated by the ubiquitination status of H2BK123 [35]. The SAGA complex component Ubp8p is a H2B deubiquitinating enzyme, that specifically removes monoubiquitin from H2BK123 [7]. This in turn modulates the level of methylation of H3K4, and hence alters the expression of Ubp8p-dependent genes, such as *GAL10* [4], [7], [34]. It has been proposed that this methyl mark may further stabilize SAGA recruitment through Chd1p interaction [12]. The observation that the Chd1p homologue is not detected as a component of the SAGA complex in the growth conditions tested in *A. nidulans* could be explained by the absence of the Ubp8p homologue from the SAGA complex. It is evident that Chd1p function in *S. cerevisiae* is dependent upon the Upb8p function. Therefore, since the homologue of Ubp8p is not detected as a component of the SAGA complex in *A. nidulans* under the growth conditions tested, it is possible that the homologue of Chd1p lost its functional requirement for the SAGA complex.

### Conclusions

Most components of the yeast SAGA complex were identified in the *A. nidulans* genome, and using a TAP-tagged version of SptC we were able to confirm that these components are in a complex in *A. nidulans*. In the conditions tested in this study, the homologues of Ubp8p and Chd1p were not detected as part of the SAGA complex in *A. nidulans*, which is a key difference between the SAGA complexes of *A. nidulans* and *S. cerevisiae*. The deubiquitinating module is present in the human SAGA complex [36]. The absence of Ubp8 and Chd1 in the complex is consistent with the absence of clear homologues of Sus1p and Sgf11p in the *A. nidulans* genome. In *S. cerevisiae*, Gcn5p HAT activity in SAGA is independent of its deubiquitinating activity [9].

Further, in was evident that there were no apparent compositional differences between acetate or glucose repressing growth conditions and non-repressing growth conditions, indicating that dynamic changes in SAGA complex composition are not important in acetate or glucose repression.

Further experimentation will confirm and determine the significance of these differences within the SAGA complex between the two organisms, and whether the proteins not identified as components of the SAGA complex in *A. nidulans* are present in other complexes that provide these functions. Our results clearly show that there are important differences between the deubiquitination networks of *S. cerevisiae* and *A. nidulans*. Interestingly, *S. cerevisiae* also lacks a clear homologue of the conserved deubiquitinating enzyme encoded by the *creB* gene in *A. nidulans*, despite clear homologues being present in insects and vertebrates [37].

## Supporting Information

Figure S1 **Confirmation of ^N−TAP^** ***sptC*** **.** A) Ampilfication of the *sptC* locus from the *A. nidulans* using primers S3KO1 and S3KO4 [21]. B) Amplified *sptC* restriction products: C:*Apa*I; U:undigested. As expected, a 2.4 kb band was amplified for the wild type strain and a 2.9 kb band for the transformant, as the N-TAP tag is 0.5 kb. The restriction enzyme *Apa*I was used to digest the amplified products. The *ApaI* recognition site is incorporated within the N-TAP tag; thus, only the amplified product from the transformed strain will be digested by the *Apa*I restriction enzyme, producing bands of 1954 bp and 946 bp. The amplified product from the wild type strain contains no ApaI site. DNA sequencing confirmed that the tag was in frame and the gene mutation free.
(DOCX)

Table S1 **Proteins identified in the** ***A. nidulans*** **SAGA complex.**
(DOCX)
