# Supplementary material for: The Spt-Ada-Gcn5 Acetyltransferase (SAGA) Complex in Aspergillus nidulans
Source: PLoS One. 2013 Jun 7;8(6):e65221. doi: 10.1371/journal.pone.0065221 (PMC3676421; doi:10.1371/journal.pone.0065221)
Supplement: Figure S1 — Confirmation of N−TAP sptC . A) Ampilfication of the sptC locus from the A. nidulans using primers S3KO1 and S3KO4 [21]. B) Amplified sptC restriction products: C:ApaI; U:undigested. As expected, a 2.4 kb band was amplified for the wild type strain and a 2.9 kb band for the transformant, as the N-TAP tag is 0.5 kb. The restriction enzyme ApaI was used to digest the amplified products. The ApaI recognition site is incorporated within the N-TAP tag; thus, only the amplified product from the transformed strain will be digested by the ApaI restriction enzyme, producing bands of 1954 bp and 946 bp. The amplified product from the wild type strain contains no ApaI site. DNA sequencing confirmed that the tag was in frame and the gene mutation free. (DOCX) [file pone.0065221.s001.docx]

**Figure S1**. Confirmation of ^N-TAP^*sptC*. A) Ampilfication of the *sptC* locus from the *A. nidulans* using primers S3KO1 and S3KO4 [21]. B) Amplified *sptC* restriction products: C:*Apa*I; U:undigested. As expected, a 2.4 kb band was amplified for the wild type strain and a 2.9 kb band for the transformant, as the N-TAP tag is 0.5 kb. The restriction enzyme *Apa*I was used to digest the amplified products. The *ApaI* recognition site is incorporated within the N-TAP tag; thus, only the amplified product from the transformed strain will be digested by the *Apa*I restriction enzyme, producing bands of 1954 bp and 946 bp. The amplified product from the wild type strain contains no *Apa*I site. DNA sequencing confirmed that the tag was in frame and the gene mutation free.
