# Supplementary material for: The Spt-Ada-Gcn5 Acetyltransferase (SAGA) Complex in Aspergillus nidulans
Source: PLoS One. 2013 Jun 7;8(6):e65221. doi: 10.1371/journal.pone.0065221 (PMC3676421; doi:10.1371/journal.pone.0065221)
Supplement: Table S1 — Proteins identified in the A. nidulans SAGA complex. (DOCX) [file pone.0065221.s002.docx]

**Table S1** Proteins identified in the *A. nidulans* SAGA complex.

| ***Complex components*** | ***Accession number*** | ***Predicted MW (kDa)*** | ***Sequence coverage (%)*** | ***Identified peptides*** |
| --- | --- | --- | --- | --- |
| Spt20p | AN0976  (RfeE) | 106.8 | 9 | FYEGCLIVR  TVAELAADEALAAEEER  VFTTVLHPTPR  FKTLENIR  SLQAELTLLATTPDPR  VIIEHIR  QHLAAQQQAQAQLAAQQQNR |
| Spt7p | AN4894 | 123.9 | 25 | GSIDADGPHTGNLISEADTVSEEDPR  NMVQASDAELR  IGQEELYEAAEK  DAPDYYNVIK  EDSEGNLVDNSEDFLRVLPK  GLFTQPDSKLSR  VPVADSVEATSSPQWKR  LFKGDKLNSDEPALLR  IALFR  QMQLQSQMYQNQFQK  SLMSEVRK  DRAEVEAEER  YNTNPEHFLRK  TFNGYPKPFSGSR  ETEKLVPLIPDIVIRDR  LPEDTLDDAVAVLEGAEAEDKR  MYNAAQNTNTSQSVTVFPPPPPYPR  KLDDDDYDDYDDDDDDDAKPASSPALK  ALIAAER |
| Ada1p | AN10953 | 45.2 | 14 | ALISVSR  EELAFTR  IDPIICADPK  DGATGLLPVEAK  AAEDSASAVPPSR  LDLGPPYLELK |
| Gcn5p | AN3621  (GcnE) | 47.1 | 10 | RGEIEFR  SHLSIAIVK  IDPLSIPAIK  IVPFPEKPAVLEER |
| Ada2p | AN10763  (AdaB) | 58.6 | 19 | MTVVDIYNTR  LAIAQLQEWR  TKEERELLNK  ILFEHNLLEYR  AQEPNVPLNDFDR  ADPEDTRLQDSISKEEFQAR  QNQQSEQPTAANQLTTPELPLRLQK |
| Ada3p | AN0440 | 72.8 | 25 | LYSLLR  LLQSELK  QMIINSAR  LLEVARER  MGGVGPIPDSK  FDDPTIYHIR  GDACNAGIRELSQK  LVIPVTIQYSEILER  EVVDEPEPYEGDDRVK  TTVPGPGESLFTPSVMAEYEK  GNIDQVTDETIETDKVSVGPLVSR  EVTPNMTDEEKKEIYSVNVFPSSDLR |
| Spt8p | AN4670  (AcdX) | 71.6 | 28 | LMLTVAQR  FLVSTSGNR  HPFVDSVIK  SLLSGSEDKR  NGTVEEYSLHK  WVFSGGSDGYVR  KFNWVDSINSK  LYDLKHEQATR  HSTVPFLIIPGHR  IYDWDLNTGQTR  TFKFPQGSGPVTALK  TGTVSQLYVDQACR  GWEGSTTEVLLGYEIGVPVVR  RVFGSSAGQISAIELRPESSLPVPR |
| Spt3p | AN0719  (SptC) | 41 | 27 | ETGLFDPPEEGR  DDEEDEEEEEQNYATLQR  GGADAADFADDPMAGGVVAGPQDVASKPK  SISTDDLIFLIR  NIAMLLHNGR  ETGLFDPPEEGRTPVEPR |
| Taf5p | AN0292 | 80.9 | 36 | IDGASSAAANSTQTPGTVGPGMGSGKK  VAGEGGAGTKIDGASSAAANSTQTPGTVGPGMGSGK  LVLSNPAFGSLMQFLESK  KIPSDDSADEPPTNSRR  MWAVTTGNAVR  LIGHSGPVYAVAFAPSATPSENAVAPTNAR  DVVVTPDQISAFPTK  WLLSSSADR  ADIPYPPSTAR  DVAIEVQKVKENR  FTNMNLIIAGGAYLP  VWDVMGPAHDPSQGR  MFTGHTGNITALACSR  ALEPISLPEHVQDNSIAK  TLASADDHGSILLWDLGPGR |
| Taf6p | AN8232 | 48.6 | 26 | IAQVLEEALK  VCNAFLDESSEKYR  TLLTTQDIALALR  ELQLYFEK  VAELGDARVAHAILG  QLGGTADLSEQFALR  VLIIPNLPIYGNLLK  VLDVEPLYGYETTRPLK  LGAFVAEKVAELGDAR  VCNAFLDESSEK |
| Taf9p | AN0794 | 31.6 | 25 | LHYQFQTGLPK  VPLQLLDFAYR  GPPEVNSVTLPALR  EFLMDVAAER  GFDAGAGKPAASNSVLMAGMR  LSIASR |
| Taf10p | AN0154 | 30 | 5 | FIADIAADSYQYAR |
| Taf12p | AN2769 | 64.7 | 33 | YGIALQK  MLDVLVR  EGSEFLAK  NVNMVIPK  VQHTQLVR  DIQLVLER  LPHLNDQQK  ISGFSTDDLR  LSQLSQSLMK  NFWEILNSR  FKEQQDLFK  LFREGSEFLAK  HPGYVLEGEGQR  YGIALQKQEIGR  DAQSPEYQQAHTR  QSAGNLTGEEMQEFKNR  SQSVNPQNFAQLLPQFQQK  QLQHQQMQAASAAQGQPVQR |
| Sgf73p | AN11747 | 43.5 | 10 | ASPATSGSGGFIEASGYK  QCGVPLPNGAQCAR  TMSVFPTGKPR  SQPQPLVTHTLIST  SLPYDMLLQAYQK  IKEMLSHALGGAR |
| Sgf29p | AN0668 | 50 | 28 | FVLDIGGK  NLGLLTALR  QASEAEAPLSR  YPDTTTFYR  TTAVSLIPIPR  IGSALPSFPVGK  ALAEHIAALNEK  AILQDEPSDVIK  STSASSTQVRDNVQVKIEEGAESAK  NDFDGSATDSPGPVGGPSDK |
| Tra1p | AN8000 | 382.5 | 2 | SPDVIDAANAGLR  DLVYNNSDIIVNR  LPAATEANITR  ALTYVIKDTPPDLPASTR  IFNGVLGK  QPDLFYESR |

Legend: Shading indicates separate functional modules.
